# Ambulatory heart rate of professional taxi drivers while driving without their typical psychosocial work stressors: a pilot study

**DOI:** 10.1186/s40557-016-0139-7

**Published:** 2016-10-06

**Authors:** BongKyoo Choi, SangJun Choi, JeeYeon Jeong, JiWon Lee, Shi Shu, Nu Yu, SangBaek Ko, Yifang Zhu

**Affiliations:** 1 Center for Occupational and Environmental Health, University of California Irvine, 100 Theory, Suite 100, Irvine, CA 92617 USA; 2 Department of Occupational Health, Catholic University of Daegu, Gyeongsan, South Korea; 3 Department of Occupational and Environmental Health, Yong In University, Yongin, South Korea; 4 Department of Family Medicine, Yonsei University College of Medicine, Seoul, South Korea; 5 Department of Environmental Health Sciences, University of California Los Angeles, Los Angeles, CA USA; 6 Department of Preventive Medicine, Yonsei University Wonju College of Medicine, Wonju, South Korea

**Keywords:** Los Angeles, Percent maximum heart rate, Physical hazards, Psychosocial hazards, Work hours, Work stress

## Abstract

**Background:**

Few studies have examined ambulatory cardiovascular physiological parameters of taxi drivers while driving in relation to their occupational hazards. This study aims to investigate and quantify the impact of worksite physical hazards as a whole on ambulatory heart rate of professional taxi drivers while driving without their typical worksite psychosocial stressors.

**Methods:**

Ambulatory heart rate (HR_driving_) of 13 non-smoking male taxi drivers (24 to 67 years old) while driving was continuously assessed on their 6-hour experimental on-road driving in Los Angeles. Percent maximum HR range (PMHR_driving_) of the drivers while driving was estimated based on the individual HR_driving_ values and US adult population resting HR (HR_rest_) reference data. For analyses, the HR_driving_ and PMHR_driving_ data were split and averaged into 5-min segments. Five physical hazards inside taxi cabs were also monitored while driving. Work stress and work hours on typical work days were self-reported.

**Results:**

The means of the ambulatory 5-min HR_driving_ and PMHR_driving_ values of the 13 drivers were 80.5 bpm (11.2 bpm higher than their mean HR_rest_) and 10.7 % (range, 5.7 to 19.9 %), respectively. The means were lower than the upper limits of ambulatory HR and PMHR for a sustainable 8-hour work (35 bpm above HR_rest_ and 30 % PMHR), although 15–27 % of the 5-min HR_driving_ and PMHR_driving_ values of one driver were higher than the limits. The levels of the five physical hazards among the drivers were modest: temperature (26.4 ± 3.0 °C), relative humidity (40.7 ± 10.4 %), PM_2.5_ (21.5 ± 7.9 *μg*/m^3^), CO_2_ (1,267.1 ± 580.0 ppm) and noise (69.7 ± 3.0 dBA). The drivers worked, on average, 72 h per week and more than half of them reported that their job were often stressful.

**Conclusions:**

The impact of physical worksite hazards alone on ambulatory HR of professional taxi drivers in Los Angeles generally appeared to be minor. Future ambulatory heart rate studies including both physical and psychosocial hazards of professional taxi drivers are warranted.

## Background

Professional taxi drivers are at high risk of coronary heart disease and stroke [1–3]. In addition, hypertension, hyperlipidemia, type 2 diabetes mellitus, and obesity are prevalent among professional taxi drivers [4–9]. However, few studies [10] have examined ambulatory cardiovascular physiological parameters of taxi drivers while driving in relation to their worksite physical or psychosocial hazards, which is essential for elucidating the etiology of cardiovascular disease (CVD), assessing the separate and combined CVD risks of worksite physical and psychosocial hazards of taxi drivers, and developing strategies for preventing CVD in taxi drivers.

This study as a pilot study is our first step toward filling the current research gap in the literature. We took advantage of our unique chance to assess ambulatory heart rate of professional taxi drivers in Los Angeles who participated in a 6-hour field exposure assessment project about air pollutant exposures inside taxi cabs [11]. During the field exposure assessment, professional taxi drivers were exposed as usual to several inside-taxi-cab physical hazards (e.g., noise and air pollutants) [10, 12], while driving on the road. But, the drivers while driving were free from taxi drivers’ typical psychosocial work hazards (e.g., conflicts with passengers, violence from passengers, searching for potential passengers, time pressure, shift work/long work hours, and traffic jams) [4, 13–15] because their time and effort were compensated by the project research fund. Thus, the field exposure assessment project naturally created an ideal situation for us to examine and quantify the unique impact of worksite physical hazards as a whole on ambulatory heart rate of taxi drivers while driving, independent of worksite psychosocial hazards. This information will substantially facilitate future risk assessments of the separate or combined contributions of worksite physical and psychosocial hazards to ambulatory heart rate of taxi drivers, which is particularly important for both prioritizing intervention areas and estimating the impact of interventions to improve the cardiovascular health (ambulatory heart rate) of professional taxi drivers.

Ambulatory heart rate during work has been used in the occupational health/ergonomic literature as a reliable indicator of cardiovascular strain of the workers who are exposed to diverse worksite physical and psychosocial hazards [13, 16–21]. In addition, 24-hr averaged ambulatory heart rate on a work day was a strong predictor for all-cause mortality after controlling for clinical resting heart rate in a recent study [22]. Pathophysiologically, elevated heart rate can affect all stages of the CVD continuum from atherosclerosis to end stage of heart failure [23]. It promotes atherosclerosis through increased vascular oxidative stress and inflammation, endothelial dysfunction, and arterial stiffness [23, 24]. Also it increases systolic time, while it decreases diastolic time [25, 26]. Thus, it results in increased myocardial oxygen consumption and decreased coronary blood flow and ventricular filling time [23–25].

The purpose of this study is to investigate and quantify the impact of worksite physical hazards as a whole, including temperature, relative humidity, noise, PM_2.5_, and CO_2_ inside taxi cabins, on ambulatory heart rate of professional taxi drivers while driving without their typical worksite psychosocial stressors in a group of taxi drivers in Los Angeles, California. Our main foci are how much ambulatory heart rate of professional taxi drivers will be elevated above their resting heart rate and whether ambulatory heart rate of professional taxi driver goes beyond the general upper limits of ambulatory heart rate recommended for a sustainable 8-hour work (i.e., 35 beats per min above the resting heart rate and 30 % of percent maximum heart rate range) [9, 27, 28].

## Methods

### Background of the field study

A total of 22 professional (21 males and 1 female) taxi drivers participated in a field exposure assessment project about air pollutant exposures inside taxi cabs [11]. The research team ran a recruitment/survey campaign at the Los Angeles Airport (LAX) taxi holding lot in February 2013 in order to recruit study participants and collect basic information about taxi drivers and their cabs. A questionnaire designed by the research team included the questions about sociodemographic, car model, work stress experience, health-related behaviors, health conditions, and the use of medication [11]. We initially handed out 2449 questionnaires and finally collected 316 complete questionnaires. Out of the 316 taxi drivers who completed the questionnaire, 121 non-smokers were eligible to participate in a field exposure assessment project about air pollutants and air exchange rates inside taxi cabs. To ensure the sampled taxi drivers/cabs are representative, stratified random sampling was conducted based on car models and drivers’ age. A total number of 22 non-smoking taxi drivers out of 121 eligible drivers were selected to participate in the field exposure assessment project [11]. The study design and protocol (#12-000845) were approved by the Institutional Review Board (IRB) of University of California Los Angeles. All of the taxi drivers who participated in the field exposure assessment project provided written informed consent.

### 13 male taxi drivers for this study

Among the above 22 taxi drivers, we excluded 9 taxi drivers from this study whose ambulatory heart rate on the first experimental day (for details, see below) was not be able to be collected (*N* = 5; cases T03, T07, T08, T19 and T20, including 1 female driver) or was collected, but only for a limited time period (<1 h) (*N* = 4; cases T05, T12, T18, and T21). Thus, we included 13 male taxi drivers in the current study. There were some differences between the excluded and included drivers: the excluded 9 drivers were older (53.0 years vs. 42.9 years), worked more years as a taxi driver (11.6 years vs. 7.4 years), and had higher prevalence of hypertension (33 % vs. 15 %) than the included 13 drivers.

### 6-hour experimental on-road driving

The 13 male drivers drove 6 h on each of four consecutive experimental days in the Greater Los Angeles area between April 2013 and November 2013 as he would typically do. One field technician rode along in the taxi cab operating and maintaining all of the air sampling instruments (see below). The starting time of each day was based on the driver’s availability. No actual fares were collected during the tests and the drivers’ time and effort were compensated by the research fund [11]. The driving routes were not specifically planned for each driver. Instead, each driver was asked to drive from the start location, University of California Los Angeles, to the area where he usually works. Each driver was allowed to take breaks as he would during a typical work day. The time and location of each break were recorded by hand by one field technician and confirmed by a GPS unit (Qstarz GPS BT-1000XT, Taipei, Taiwan). The experimental conditions with regard to taxi-cab air flow varied day to day during the four experimental days [11]. The experimental conditions on the first day were least intervening (most realistic) because the drivers had a full control over all the vehicle operations such as opening/closing windows, turning air conditioning on or off, setting ventilation to recirculation or outdoor air mode as they usually did on their typical work days. Thus we restricted our analyses for this study to the first experimental day.

### Assessment of ambulatory heart rate: HR_driving_, HR_rest_, and PMHR_driving_

One trained field technician instructed the drivers to wear and use an ambulatory heart rate monitor (RS800CX, Polar Electro, Finland, with a sampling rate of 1,000 Hz) [29, 30] just before the experimental 6-hr on-road driving. Heart rate (HR) of the drivers was continuously measured based on the peak to peak interval of two consecutive QRS complex signals on electrocardiograms (called hereafter RR interval, milliseconds) with the monitor during the driving. The raw RR interval data of 13 drivers downloaded from the monitor were processed using the data analysis software (Kubios Version 2.2) [31] with the medium option for artifact correction. Then, we split the artifact-corrected RR interval data into 5-min segments (674 segments in total) from the starting time of the 6-hr driving and calculated the average HR of each 5-min segment (called hereafter 5-min HR). Afterwards, we manually examined each 5-min segment whether all RR intervals in the 5-min segment are within the normal range (300 to 2,000 milliseconds) and whether most (>95 %) of the ratios of two consecutive RR intervals in the 5-min segment are within the normal range (0.8 to 1.2) [30, 32]. Of the total 5-min 674 segments, 160 (23.70 %) segments were excluded by the last procedure from analyses in the current study.

After the above data cleaning process, each 5-min segment was classified into the following two groups: 5-min HR while driving (5-min HR_driving_) and 5-min HR while taking breaks (5-min HR_breaking_) based on the record by one field technician on the on-road driving. In the current study, we used only 5-min HR_driving_ values (of 344 5-min segments in total) for analyses as consistent with the previous environmental science studies in taxi drivers [10, 11]. The driving times noted by one field technician were confirmed by matching the driving times with the vehicle speed information (i.e., ≥ 1 km∙hr^−1^) during the same time periods. The breaking times recorded by the field technician included the times when the drivers took a rest inside taxi cabs, or went outside taxi cabs for a stretch, walk, or meal.

Resting HR (HR_rest_) of each taxi driver was estimated conservatively based on the following two-step process: (1) identifying the lowest 5-min HR_driving_ value of each driver [28]; and then (2) choosing the age- and gender-specific 25th, 50th, or 75th HR_rest_ percentile value in the United States (US) adult population reference data from the 1999–2008 National Health and Nutrition Examination Surveys [33] that was lower than, but closest to the identified lowest 5-min HR_driving_ value.

As a way to control for the individual differences in age and resting and maximum HR, we estimated the 5-min percent maximum heart rate range (PMHR_driving_) of each taxi driver during the driving times using the following equation [9]:

$$ 5\hbox{-} \min\ {\mathrm{PMHR}}_{\mathrm{driving}}\left(\%\right) = \frac{5 \min {\mathrm{HR}}_{\mathrm{driving}}-{\mathrm{HR}}_{\mathrm{rest}}}{{\mathrm{HR}}_{\max }-{\mathrm{HR}}_{\mathrm{rest}}} \times 100 $$

The maximum heart rate (HR_max_) of each driver was estimated by using the formula, 205.8 − (0.685 × age) [34, 35].

### Assessment of five physical (environmental) hazards while driving inside taxi cabs

Five physical hazards (PM_2.5_, CO_2,_ relative humidity, temperature, and noise) were continuously assessed inside taxi cabs during the experimental 6-hr on-road driving. One DustTrak (Model 8520, TSI Inc., St. Paul, MN) was used to measure the in-cabin PM_2.5_ concentrations. One Q-trak monitor (Model 8554, TSI Inc., St. Paul, MN) was also used to measure the in-cabin CO_2_ concentrations, relative humidity, and temperature simultaneously. The noise level in cabin was measured by a Quest 2900 Sound Level Meter (3 M, St. Paul, MN). All of these instruments were synchronized with the heart rate monitor and were set to record one reading every second, to provide data with high time resolution. All instruments were calibrated to ensure the quality of measurement. After the experimental driving, data were downloaded. Data were then observed in Excel for visualization and obvious outliers - mostly caused by instruments malfunctioning - were removed before further data analysis. Then as with the HR data, the physical hazards data were also split and averaged into 5-min segments for analyses and only the 5-min averaged data while driving (excluding the 5-min averaged data while breaking) was used in the current study.

### Assessment of work hours, work stress, and other covariates

Work hours per week were calculated using the two questions in the recruitment survey questionnaire (“Typically, when do you start and end your work day?” and “How many days do you typically work as a taxi driver each week?”). Work stress experience of taxi drivers on a typical workday was also measured with one question (“How often do you find your work stressful?). Age, race/ethnicity, exercise during leisure-time (the frequency of the moderate or vigorous level of aerobic exercise: frequent (2 or more times per week) and infrequent (0–1 times per week))), health condition (“In general, would you say your health is: Excellent; very good; good; fair; and poor), body weight and height, hypertension (“Have you ever been diagnosed with hypertension?”), and the use of anti-hypertensive medication were also assessed with the recruitment survey questionnaire.

### Data analysis

At first, the distributions of five physical hazards inside taxi cabins during the 5-min HR_driving_ periods for each driver were examined. Then we examine the distribution (maximum, mean, and minimum) of the 5-min HR_driving_ values for each driver with a focus on the extent of the elevation of HR and the threshold HR value for a sustainable 8-hour work (i.e., 35 beats per min above HR_rest_) [9]. The above analyses were replicated with the 5-min PMHR_driving_ values among the 13 taxi drivers with a focus on the threshold PMHR value for a sustainable 8-hour work (i.e., 30 % of percent maximum heart rate range) [27, 28]. As a sensitivity test, we tested whether the above analyses would be affected by the status of hypertension, obesity, and exercise frequency of the taxi drivers. All data analyses and graphs were performed using PASW version 23.0 (SPSS Inc., Chicago, IL, USA) and Sigma Plot software, version 12.5 (Systat Software Inc. USA), respectively.

## Results

### Sociodemographic and work environmental characteristics of the 13 male taxi drivers

The mean age of the taxi drivers was 42.9 years (range: 24–67 years). Their mean career years as a taxi driver were 7.4 years (range: 0.5–17 years). They were mostly Asian or Black (Table 1). Their self-reported health was mostly good or very good. Four out of the thirteen taxi drivers reported that they engaged in moderate or vigorous level of leisure-time aerobic exercise 2 or more times per week. The average body mass index (BMI) of taxi drivers was 26.4 kg/m^2^ and three out of them (23 %) had greater than BMIs of 30 kg/m^2^. The drivers worked, on average, about 12 h (range, 9 to 14 h) on a typical work day, 6 days per week (range 5 to 7 days), and 72 h (range, 54 to 98 h) per week. Seven (53.8 %) drivers reported that their job was often stressful on typical work days, while six drivers reported that their job was sometimes or hardly ever stressful on typical work days. Two (15 %) drivers had hypertension and only one driver was under treatment of anti-hypertensive medication at the time of the questionnaire survey.

**Table 1:** Physical hazards and ambulatory heart rate (HR_driving_) and percent maximum heart rate range (PMHR_driving_) while driving in the 13 male taxi drivers

Case	Physical hazards inside taxi cabs					Vehicle speed (km/h)	5-min HR_drving_ (bpm)				^c^HR_rest_(bpm)	^d^HR_max_(bpm)	5-min ^e^PMHR_driving_(%)
	Temperature (°C)	Relative Humidity (%)	Noise (dBA)	PM_2.5_(μg/m^3^)	CO_2_(ppm)		# of 5-min HR values	Mean	Max.	Min.			Mean
T01	24.1	38.8	72.2	16.7	1042.7	47.3	46	79.0	89.6	72.7	69 (50th)	189.4	8.3
T02	22.1	36.5	68.4	8.9	917.3	29.5	38	89.2	101.9	81.2	78 (75th)	185.9	11.9
T04	32.2	31.3	68.6	22.8	1522.3	44.7	20	74.7	85.1	63.4	61 (25th)	186.6	11.0
T06^a^	24.3	53.4	70.5	33.1	684.1	32.4	22	73.7	80.4	70.7	68 (50th)	170.9	5.7
T09	28.4	32.5	68.2	14.8	1756.1	24.8	26	86.3	112.5	71.8	68 (50th)	173.6	19.9
T10	31.3	45.7	69.9	30.8	499.7	38.7	16	83.3	94.6	73.7	67 (50th)	159.9	14.6
T11	27.2	36.6	73.6	13.3	1661.5	69.2	35	79.5	86.9	72.0	68 (50th)	177	9.0
T13	25.5	61.1	68.9	21.2	1971.3	37.8	32	69.3	74.1	65.7	61 (25th)	166.8	7.5
T14	26.4	42.3	73.2	32.8	1139.8	48.6	16	70.0	80.7	63.4	61 (25th)	183.9	8.3
T15	27.6	26.3	69.4	14.8	2499.3	38.2	22	86.3	94.1	81.6	77 (75th)	168.1	10.0
T16	24.5	36.7	70.2	19.6	832.9	52.5	28	86.2	102.7	78.6	77 (75th)	168.8	9.8
T17	26.6	32.2	61.4	21.3	769.3	23.2	17	90.3	98.3	84.0	78 (75th)	186.6	13.8
T22^b^	23.1	55.2	70.7	28.6	1176.3	51.1	26	78.0	88.4	71.8	68 (50th)	175.7	9.5
Mean	26.4	40.7	69.7	21.5	1267.1	41.4	26.5	80.5	91.5	73.1	69.3	176.4	10.7
SD	3.0	10.4	3.0	7.9	580.0	12.7	9.2	7.1	10.6	6.7	6.4	9.4	3.7

Age [mean (range)]: 42.9 (24–67) years. Driving career [mean ± SD]: 7.4 ± 5.4 years. *bpm* beats per minute, *Max* maximum, *Min* minimum, *SD* standard deviation. ^a^Hypertensive without taking anti-hypertensive medication. ^b^Hypertensive under treatment with anti-hypertensive medication. ^c^HR_rest_: the age-and gender(male)-specific 25th, 50th or 75th HR_rest_ percentile in the US adult population reference data from the 1999–2008 National Health and Nutrition Examination Surveys [33]. ^d^HR_max_: based on the equation, 205.8 − (0.685 × age). ^e^ ${\mathrm{PMHR}}_{\mathrm{driving}}=\frac{\left({\mathrm{HR}}_{\mathrm{drving}} - {\mathrm{HR}}_{\mathrm{rest}}\right)}{\Big({\mathrm{HR}}_{\max } - {\mathrm{HR}}_{\mathrm{rest}\Big)}} \times 100$

### Distributions of in-cabin environmental physical hazards among 13 male drivers

The means and standard deviations of the five in-cabin physical hazards while driving were as follows: temperature (26.4 ± 3.0 °C), relative humidity (40.7 ± 10.4 %), PM_2.5_ (21.5 ± 7.9 *μg*/m^3^), CO_2_ (1,267.1 ± 580.0 ppm) and noise (69.7 ± 3.0 dBA). The means of the physical hazards for each driver were presented in Table 1.

### Distributions of the 5-min HR_driving_ values

**Fig. 1:**
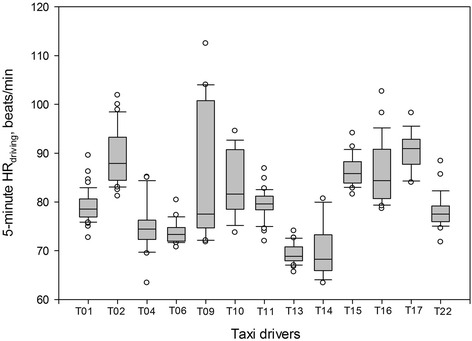
Distribution of the 5-min HR (HR_driving_) values for each of the 13 taxi drivers (T01 to T22) while driving on his 6-hr experimental on-road driving

The number of 5-min HR values of each driver on the 6-hr experimental on-road driving ranged from 16 (90 min in total) to 46 (230 min in total): on average, 26.5 (132.5 min in total) among the drivers (Table 1). Among the 13 drivers, the mean of 5-min HR_driving_ values was 80.5 bpm (range, 73.7 to 86.3 bpm). It was 11.2 bpm higher (*p* < 0.001) than the mean of the estimated HR_rest_ values, 69.3 bpm. In 1 out of the 13 drivers, 4 of the 26 (15.4 %) 5-min HR_driving_ values were 35 bpm higher than his HR_rest_ value (Table 1 and Fig. 1). There were no significant differences in the mean of 5-min HR_driving_ values between normotensive and hypertensive drivers: 81.3 bpm and 75.9 bpm, respectively; between non-obese and obese drivers: 80.3 bpm and 81.1 bpm, respectively; and between drivers who reported frequent exercise and drivers who reported infrequent exercise: 78.1 bpm and 81.5 bpm, respectively.

### Distributions of the 5-min PMHR_driving_ values

**Fig. 2:**
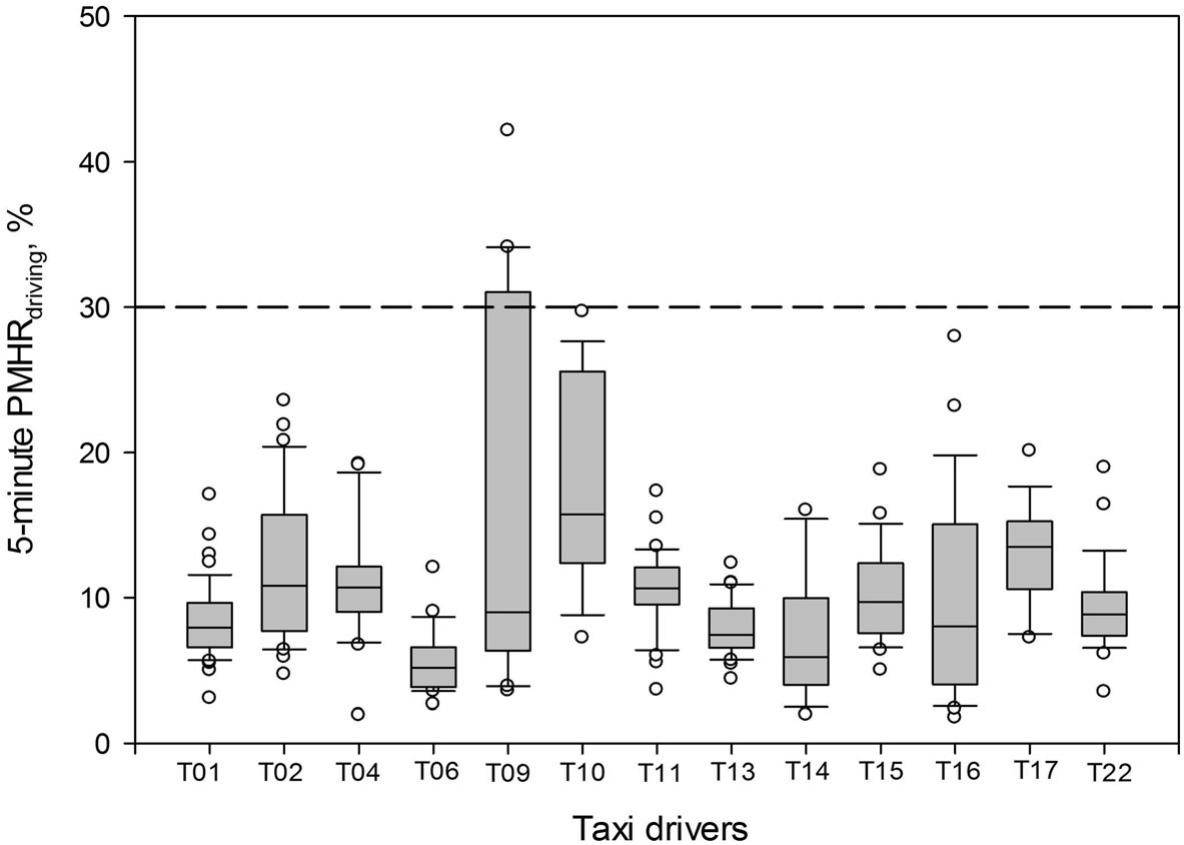
Distribution of the 5-min percent maximum heart rate range (PMHR_driving_) values for each of the 13 taxi drivers (T01 to T22) while driving on his 6-hr experimental on-road driving. The dotted line indicates the 30 % PMHR_driving_ value of T09

In general, the results of the 5-min PMHR_driving_ values were similar to those of the 5-min HR_driving_ values. There was a strong correlation between the two parameters among the drivers (Spearman rho = 0.77, *p* < 0.01). Figure 2 shows the distribution of the 5-min PMHR_driving_ values of each driver on the experimental driving. In 1 out of the 13 drivers, 7 of the 26 (26.9 %) 5-min PMHR_driving_ values were greater than his 30 % PMHR value (Fig. 2). The mean of the 5-min PMHR_driving_ values was 10.7 % (range, 5.7 to 19.9 %) among the 13 drivers. There were no significant differences in the mean PMHR_driving_ between the normotensive and hypertensive drivers: 11.3 and 7.6 %, respectively; between non-obese and obese drivers: 10.9 and 10.1 %, respectively; and between drivers who reported frequent and drivers who reported infrequent exercise: 10.5 and 10.8 %, respectively.

## Discussion

To the best of our knowledge, this is the first study that examined ambulatory HR of professional taxi drivers while driving without psychosocial work stressors in a 6-hour field environmental exposure assessment project. The levels of five in-cabin physical hazards assessed while driving were below the contemporary standard exposure limits. The means of the ambulatory 5-min HR and PMHR values of 13 professional taxi drivers were 80.5 bpm and 10.7 %, respectively. The means did not exceed the upper limits of ambulatory HR and PMHR for an 8-hour work (35 bpm above HR_rest_ and 30 % PMHR), although some of the 5-min HR (15 %, 4 out of 26) and PMHR (27 %, 7 out of 26) values of one driver while driving exceeded the upper limits. The means did not vary by the status of hypertension, obesity, and exercise among the 13 professional taxi drivers. This study suggests that the impact of physical worksite hazards alone on ambulatory HR (cardiovascular strain) of professional taxi drivers may not be substantial. The drivers worked on average 72 h per week and more than half of them reported that their job were often stressful. Future ambulatory HR studies including both physical and psychosocial hazards of professional taxi drivers are needed.

The average ambulatory HR of the professional taxi drivers while driving in the current study were 80.5 bpm (11.2 bpm higher than the average resting HR). This is similar to the previous study [27] in which the average ambulatory HR of 17 healthy municipal transportation workers were about 80 bpm.

However, it should be reminded that as mentioned before, the taxi drivers in the current study on an experimental on-road driving were free from diverse psychosocial work stressors of professional taxi drivers [4, 13–15]. The current study indicates that most professional taxi drivers work long hours and are under stress on their typical work days, which is consistent with the previous study with a larger sample of professional taxi drivers in Los Angeles [15]. Hayashi et al. [17] showed that long hours of overtime, compared to short hours of overtime, increased ambulatory 24-hr HR by 5 bpm in a group of healthy white-collar workers. In addition, the ambulatory HR of a machine operator increased up to about 20 bpm when the machine operator was frustrated due to 12 machine jams in a 25-minute period [9]. Thus, we think that the level of the ambulatory HR of professional taxi drivers on a real on-road driving in which they are exposed to not only physical hazards, but also psychosocial hazards would be greater than the level found in the current study.

The quantified contribution of physical hazards as a whole to ambulatory HR of taxi drivers in the current study (i.e., 10.7 % PMHR while driving without psychosocial work stressors) will function as a good basis for futures studies of estimating the contributions of psychosocial hazards alone or combined with physical hazards to cardiovascular strain in professional taxi drivers. The mean of in-cabin PM_2.5_ concentrations was lower in current study than in the previous study with Beijing taxi drivers [10]. The means of in-cabin relative humidity, temperature, and noise in the current study were similar to those of the previous studies in Beijing, China [10] and Delhi, India [12]. The means of in-cabin noise, CO_2_, and PM_2.5_ concentrations in the current study were lower than the current standard (8-hour) exposure limits of the hazards [36]. On the other hand, it should be reminded that our results do not mean null associations between in-cabin environmental hazards and ambulatory HR and PMHR in taxi drivers. We plan to conduct a future study in which a short-term (e.g., 5-min) change in in-cabin environmental hazards is associated with a short-term change in ambulatory HR and PMHR in taxi drivers on the 6-hr experimental on-road driving.

### Limitations

This study has three main limitations. First, the sample size was small. Future studies with a larger sample of taxi drivers, assessing both worksite physical and psychosocial hazards, will be needed to confirm the findings of the current study. However, our sample of taxi drivers in Los Angeles were comparable with a larger sample (*N* = 309) of Los Angeles taxi drivers in the study by Wang and Delp [15]. The two samples were very comparable in terms of age, years of driving, race/ethnicity, work hours per week, and work stress level. Second, we had to estimate resting HR of each driver using the two-step process in the current study. It would have been a better approach to measure resting HR of each driver before starting the experimental on-road driving. Nonetheless, we think that our estimation process is better than the previous approaches by other investigators: assigning a single resting HR (60 bpm) to all male subjects [37] and/or identifying the lowest HR during work [28]. The former ignores the individual difference in rest HR. The latter may overestimate resting HR of each subject. Our two-step approach was an effort to overcome those disadvantages using the individual lowest HR_driving_ value and the age-and gender-specific resting HR value of US adult population. In addition, our results with HR_driving_ values that had no direct relationship with the estimated resting HR were also similar to those with PMHR_driving_ values based on the estimated resting HR values. Thus, we think the bias due to the use of estimated HR values, if any, would be minor in the current study. Rather, this study provides a good method for estimating the PMHR of workers at work when their resting HR values are not able to be measured or not available. Third, we conducted a sensitivity test to see whether the results in the current study would be affected by the status of hypertension, obesity, and exercise frequency among the 13 taxi drivers. It appeared that the means of HR_driving_ and PMHR_driving_ were similar whether the drivers were hypertensive or not, obese or not, and exercised frequently or not. However, it should be reminded that all information on hypertension, obesity, and exercise were self-reported in the current study and its sample size of the current study was small as a pilot study. Furthermore, the current study was not designed to investigate the potential differences in the HR_driving_ and PMHR_driving_ by the status of hypertension, obesity, and health-related behaviors (e.g., exercise, smoking, and alcohol consumption) among professional taxi drivers, which is one of the understudied topics among taxi drivers [14, 38] and working populations [39]. For the purpose, future studies are warranted in a larger sample of professional taxi drivers with objective measures of blood pressure, obesity, and physical activity.

## Conclusions

The impact of physical worksite hazards (inside-taxi-cab temperature, relative humidity, noise, PM_2.5_, and CO_2_) on ambulatory HR of professional taxi drivers while driving without being exposed to their typical psychosocial work stressors generally appeared to be minor. More ambulatory HR studies including both physical and psychosocial hazards of professional taxi drivers are warranted for both clarifying the etiology of CVD and developing and prioritizing CVD prevention strategies in taxi drivers.
